# Effect of high hydrostatic pressure on aroma components, amino acids, and fatty acids of Hami melon (*Cucumis melo* L. var. reticulatus naud.) juice

**DOI:** 10.1002/fsn3.1406

**Published:** 2020-02-10

**Authors:** Longying Pei, Jie Li, Zhenli Xu, Nan Chen, Xiaoxia Wu, Jiluan Chen

**Affiliations:** ^1^ Department of Food Science and Engineering Xinjiang Institute of Technology Aksu China; ^2^ Food College Shihezi University Shihezi China

**Keywords:** amino acids, aroma components, fatty acids, Hami melon, high hydrostatic pressure

## Abstract

The changes and relationships between the volatile compounds and fatty acids, and between volatile compounds and free amino acids were analyzed after they were handled by 400 and 500 MPa (45°C/10 min) high hydrostatic pressure (HHP). The volatile components of 31, 30, and 32 were detected in the untreated, 400, and 500 MPa samples, respectively. Unlike the ketones and acids, the three contents, including ester (59.59%–71.34%), alcohol (5.95%–7.56%), and aldehyde (0.36%–1.25%), were greatly changed. While HHP treatment exerted a few effects on the contents of 12 kinds of fatty acids. With the increase in pressure, the contents of palmitic acid, linolenic acid, and α‐linolenic acid were remarkably reduced. The correlations between flavor compounds and amino acids, and between flavor compounds and fatty acids were studied by Pearson's correlation analysis and visualized with using the corrplot package in R software. The analysis showed that the amino acids were positively correlated with (*E*)‐6‐nonenal, (2*E*,6*Z*)‐nona‐2,6‐dienal and (*Z*)‐6‐nonen‐1‐ol, while they were negatively correlated with nonanal, (*Z*)‐3‐hexen‐1‐ol and ethyl caproate. Besides, the fatty acids were positively correlated with the esters of 2,3‐butanediol diacetate and 2‐methyl propyl acetate, while they were negatively correlated with (*E*)‐2‐octenal and (*Z*)‐6‐nonen‐1‐ol.

## Graphical abstract

The changes and relationships between the volatile compounds, fatty acids, and free amino acids of Hami melon juice, treated by high hydrostatic pressure.

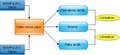

## 1 INTRODUCTION

Hami melon (*Cucumis melo* L. var. reticulatus naud.) is also called cantaloupe. Xinjiang Uygur Autonomous Region serves as a major producing region of Hami melon in China because of its strong sunshine and the typical continental climate. Benefited from the favorable representative desert climate, the melons produced in Xinjiang were not only juicy, crisp, sweet, and aromatic, but also abundantly nutritional. Hami melon was a thermally sensitive fruit (Pang, Chen, Hu, Zhang, & Wu, 2012), which obviously affected its quality and industrial development. Therefore, studying the effects of new nonthermal processing technologies on the aroma‐active compounds, free amino acids, and fatty acids of Hami melon can provide important references to improve its commercial value.

High hydrostatic pressure has been widely applied in processing food for the past decade. This technology could inactivate spores, pathogenic microorganisms, and enzymes under certain pressure, temperature, and time process, so as to increase the shelf life of food (Nienaber & Shellhammer, 2010). Furthermore, high hydrostatic pressure (HHP) better preserved bioactive and aroma compounds of most foods than thermal processing, such as vitamins, carotenoids, phenolics, esters, and ketones (San Martín, Barbosa‐Cánovas, & Swanson, 2002).

Aroma is the fundamental quality criteria of fruits in the aspect of consumer acceptance. There are numerous varieties of flavor components which are identified by distinct combinations of active aroma compounds of the fruit juice. Cultivar and physiological feature of fruit play a crucial role in determining the aromatic nature. The previous researches primarily focused on the flavored substances' changes of Hami melon. The researches conducted by Pang et al. (2012) utilized GC–mass spectrometry (GC‐MS) and headspace solid‐phase microextraction (HS‐SPME) to study active aroma compounds of Jiashi muskmelon juice. In addition, another study identified 27 volatile aroma compounds (one terpene, one sulfur compound, seven alcohols, eight aldehydes, and 10 esters) in the growing and ripening stages of Hami melon (Su, Lim, Hong, & Lee, 2011). Moreover, Chen, Ning, Wang, and Wang (2011) adopted the SPME/GC–MS method to extract the aroma components from “Golden Phoenix” Hami melons and detected more than 30 volatile compounds, ranging from esters, alcohols, aldehydes to aromatic compounds. However, literatures of these researches were mainly about the qualitative analysis of volatile compounds of the melons and identification of active fragrance components, but relative studies about the impacts of various nonthermal processing technologies on aroma were fewer.

Conversely, examination of the aroma compounds of HHP‐treated strawberry coulis by GC–MS was to prove the changes of aromatic compounds after HHP treatment at 800 MPa/20°C/20 min (Lambert, Demazeau, Largeteau, & Bouvier, 1999). Bermejo‐Prada, Vega, Pérez‐Mateos, and Otero (2015) implemented a research to explored hyperbaric storage at room temperature how to affect aromatic volatiles of raw strawberry juice. In addition, mandarin juices were processed by HHP (0–500 MPa) and tested by electronic nose (Qiu, Wang, & Du, 2017). The data of E‐nose were analyzed by local preserving projection that was compared with principal component analysis (PCA) as well as linear discriminant analysis to improve its diagnostic accuracy (Qiu et al., 2017).

In some literatures, esters, ketones, and alcohols were regarded as important factors that impacted the typical aroma of ripe fruits (Viljanen, Lille, Heiniö, & Buchert, 2011), and free amino acids and unsaturated fatty acids were viewed as prime precursors of aromatic synthesis in many fruits (Porretta, Birzi, Ghizzoni, & Vicini, 1995). Therefore, there was a certain connection among aroma components and the amino acids and fatty acids (Buttery, 1981; Contreras, Tjellström, & Beaudry, 2016; Peterson & Reineccius, 2002). It was reported that catalysis of oxidation of unsaturated fatty acids (i.e., linoleic and linolenic acids) by the enzymes could produce hexanal (Cremer & Eichner, 2000; Porretta et al., 1995). The amino acids had great impacts on the fruit flavor and the browning reaction (Dos‐Santos, Bueso, & Fernández‐Trujillo, 2013; Esteve & Frígola, 2007). The literatures above indicated the volume added amino acids and the formation of isoamyl acetate, total esters, and 2‐phenylethyl acetate were directly propotional, while this added amount and the synthesis of ethyl 3‐hydroxybutyrate and diethyl succinate were inversely proportional (Esteve & Frígola, 2007). For the alcohols (except 2‐phenylethanol and tyrosol), the formation of them had no indirect relation with the volume added amino acids (Dos‐Santos et al., 2013). The addition of amino acids was favored for the formation of the total acids (Garde‐Cerdán & Ancín‐Azpilicueta, 2008). However, the report has been insufficient about the correlation between amino acids, fatty acids, and aroma compounds until now.

According to our preliminary experiments (Chen, Zheng, Dong, & Song, 2013; Pei, Hou, Wang, & Chen, 2018), Hami melon juice could maintain a better quality after processing by HHP at 400 MPa/45°C/10 min and 500 MPa/45°C/10 min. In these experiments, changes of fatty acids, amino acids, and aromatic compounds caused by HHP were determined, and the correlations between fatty acids and the aromatic compounds, and between the aromatic compounds and the free amino acids were explored.

## 2 MATERIALS AND METHODS

### 2.1 Chemicals

The four chemicals including heptanol (chromatographic grade), methanol (chromatographic purity), ethanol (chromatographic grade), and *n*‐heptane (chromatographic purity) were purchased from Sigma‐Aldrich. Besides, amino acid standard solution, including 16 individual amino acid standards (solid, purity ≥98%), single fatty acid methyl ester standards, and a standard mixture of fatty acid methyl esters, was purchased from China's J&K Chemical Ltd. And other reagents were bought from Beijing Chemical Reagent Co., Ltd.

### 2.2 Preparations of melon samples and melon juice

Late maturing Jiashi muskmelons growing in Jiashi county of Xinjiang Uygur Autonomous Region, China, were harvested by the experienced melon farmers in late August 2017. Apart from the best eating quality, they were selected based on other several harvesting indexes, such as dark green skin, rich aroma, about 75% dry and firm netting skin, peduncle with a ring, and no decay or no quality deterioration. Moreover, harvested melons were immediately precooled by refrigerators under 4°C for 24 hr, followed by the conveyance to Shihezi University (Shihezi city, Xinjiang Uygur Autonomous Region, China), and they were stored in refrigerators before being analyzed.

In accordance with the method of Ma et al. (2007), the melons were washed under clear tap water; then calyx sections, pedicels, seeds, and peels were removed. Next, these melons were cut into pieces (about 1 × 2 × 3 cm^3^), thoroughly mixed, and frozen in liquid nitrogen barrels (30 L) for about 5–7 min. Finally, the pieces of melons were sealed in vacuum and sterile laminated compound bags (20 × 15 cm^2^) and then stored at −18°C. And the melons' pieces were thawed overnight in 4°C refrigerators. Bags containing melon pieces were selected randomly to be juiced through a Philips HR2838 juicer for analysis. The acquired melon juice was filtered by using an eight‐layer sterile gauze, vacuumized, sealed in sterilized laminated compound bags, and stored at 4 ± 1°C.

### 2.3 HHP treatment

High hydrostatic pressure processing equipment (Baotou Kefa High Pressure Technology Co., Ltd.) with maximum operating pressure and capacity of 600 MPa and 6 L, respectively, was respectively adopted to pressurize the Hami melon juice samples. The time to reach the designated pressure was <10 s, and the time of pressure release was <5 s. Before pressurization, deionized water was heated to the required temperature of 45°C, as the pressure transmitting medium. The samples were subject to the treatment under 400 and 500 MPa, respectively, at 45°C for 10 min.

### 2.4 Extraction of volatiles

After the high‐pressure processing, the volatiles were extracted from the samples by HS‐SPME. Three types of samples were extracted for three times by using SPME (purchased from Supelco, Inc.) after preprocessing 50/30 μm carboxen/divinylbenzene fiber or polydimethylsiloxane (PDMS) fiber at 250°C for 30 min. To avoid browning and minimize the loss of volatiles, 8 ml melon juice was transferred quickly to a headspace bottle (15 ml) containing 2.1 g NaCl, and then, the juice was equilibrated through a hot plate/laboratory stirrer (PC‐420, Corning, Inc., Life Science) at 40°C for 10 min. The volatile components in the headspace were extracted at 40°C for 30 min by a stable flex PDMS fiber placed at 1 cm away from the liquid surface under 250 rpm/min magnetic stirring. Every one of the three analytical samples was experimented for three times, respectively.

### 2.5 GC–MS analysis

After extraction, GC–MS analysis was carried out through a GC (Agilent 7890 Series, Agilent Technologies) coupled with a triple‐axis detector (Agilent 5975 Inert XL MSD, Agilent Technologies) and a DB‐5 column (30 m × 0.25 mm i.d. × 0.25 μm; Agilent Technologies). The SPME fiber head was inserted into the sample hole at 250°C with desorpting for 1 min, running time for 30 min. And fiber head was maintained 5 min to remove impurity in the sample hole, and this hole employed no crack (hole) mode. The flow rate of the carrier gas (99.9995% He) was 40 cm/s. The temperature program of the GC oven was kept at 50°C for 1 min, then was raised to 100°C at 5°C/min, and increased to 250°C at the rate 10°C/min, and it was kept for 9 min. Helium at 1.2 ml/min acted as the carrier gas, and splitless injection mode was adopted. The quadrupole mass filter and the ion source were at 106 and 200°C, respectively. The mass spectrometer was used at 70 eV in the electron ionization mode, and the total ion current in the scanning range of 33–350 m/z was monitored to record the chromatograms. The NIST library was used for preliminary identification and combined with the retention time, the actual components, and the retention index to further determine most of the components.

### 2.6 Analysis of free amino acids

An amino acid analyzer (L‐8900, Hitachi) was adopted to analyze the amount of amino acids in the samples of Hami melon juice according to the Chinese Standard GB/T 5009.124‐2016 (2016). After being hydrolyzed into free amino acids by HCl, the proteins of the juice samples were derivatized with ninhydrin solution, separated on an ion‐exchange column, and recorded spectrophotometric absorbance detection at 440 or 570 nm. To identify the amino acids (g/100 g), the retention time of these amino acids was measured and then compared with that of the standard mixed solution.

### 2.7 Fatty acid analysis

The third method of the Chinese Standard GB/T 5009.168‐2016 (2016) was used to determine the fatty acids. For total lipid extraction, 50 ml of sample was mixed with 10 ml of 95% ethanol. And the mixed liquid was transferred by a separatory funnel containing 50 ml ethyl ether/petroleum ether mixture and shaken for 5 min, and then it was left steady for 10 min. The ether layer extract was collected into a 250‐ml flask and concentrated to dryness by rotary evaporation, so as to obtain the fat extract. Eight milliliter and 2% NaOH–methanol solution was added to the fat extract. The total fatty acids were converted into fatty acid methyl esters, and nitrogen stream was utilized to dry the organic phase. Next, 20 ml *n*‐heptane was added and shaken for 2 min, and then, saturated aqueous NaCl was added. After leaving the mixture to stand for stratification, about 5 ml of the upper heptane extraction solution was pipetted into a 25 ml test tube. Approximately 3–5 g of anhydrous sodium sulfate was added, then shaken for 1 min, and stood for 5 min, before pipetting the upper phase of the solution into the sample bottle.

A Shimadzu GC‐2010 equipped with a capillary column (100 m length × 0.2 μm film thickness × 0.25 mm i.d.) was adopted for fatty acid analysis. The temperatures of the detector and the sample injector were set at 280 and 270°C, respectively. The heating procedure was as the follows: started temperature at 100°C, kept for 13 min; raised to 180°C at 10°C/min, held for 6 min; increased to 200°C at 1°C/min, maintained for 20 min; and then raised to 230°C at 4°C/min, and kept for 10.5 min. The fatty acid results were expressed as g/100 g.

### 2.8 Statistic analysis

Every experiments were conducted in triplicate, and the data analysis was analyzed, by using software SPSS (Statistical Program for Social Science) 17.0 purchased from Chicago, IL, USA. One‐way analysis of variance (ANOVA) was used to evaluate the data. The correlation was analyzed by Pearson's correlation and visualized by using the corrplot package in R (Gu, Eils, & Schlesner, 2016; Wei & Wei, 2017). The PCA was performed with employing R package ade4 (Dray & Dufour, 2007). Figures were prepared by using Origin 8.5 (MicroCal Software, Inc.).

## 3 RESULTS AND DISCUSSION

### 3.1 Change in volatile flavor compounds

The processed Hami melon juices under different high pressure processed were analyzed to decipher the resultant effects on the major aroma substances. The Hami melon juice odor was mostly consisted of various esters, alcohols, aldehydes, acids, and ketones (Chen et al., 2010). Table 1 listed the main odor compounds as well as their odor description. Ethyl acetate (intense, fruity) was an important aroma substance in Hami melon. The other substances were largely represented by various fruit odors, but ethyl 2‐methylbutanoate, heptanal, (2*E*,6*Z*)‐nona‐2,6‐dienal, and (*Z*)‐3‐hexen‐1‐ol showed cantaloupe‐like flavors (Lambert et al., 1999). And the Table 2 presented the volatile flavor compounds in the control and processed Hami melon juice. Thirty‐one volatile compounds were identified in fresh juice, whereas 30 and 32 volatile compounds were identified in the 400 and 500 MPa HHP‐treated Hami melon juices, respectively. No significant differences could be seen in the ketones, and acids between HHP and untreated samples were observed.

**Table 1:** Description of main volatile compounds in Fruits^a^

Volatile compound	Odor description
Ethyl acetate	Ether odor, fruity
Propyl acetate	Pear, strawberry, fruity
Ethyl 2‐methylpropanoate	Fruity, sweet
Ethyl butanoate	Strong fruity
Ethyl 2‐methylbutanoate	Apple, green, cantaloupe‐like, fruity, melon
2‐methyl butyl acetate	Fruity
Ethyl caproate	Fruity, apple, wine
Hexyl acetate	Apple, pear, fruity
(Z)‐3‐hexen‐1‐ol	Grass
Ethanol	Soft, sweet wine aroma
Heptanal	Fat, citrus
Decanal	Diluted with sweet orange, fat
Nonan‐1‐ol	Citrus, fat
Nonanal	Fat, citrus, green
(E)‐non‐6‐enal	Melon, wax, green, honeydew melon, fruity
(2E,6Z)‐nona‐2,6‐dienal	Cucumber‐like, green, melon
(Z) ‐6‐nonen‐1‐ol	Melon, wax, green, and fat
(E)‐2‐nonenal	Fat, diluted with flower green incense

a Odor descriptions adapted from Viljanen et al. (2011) and Lambert et al. (1999).

**Table 2:** The changes of volatile flavor compounds in Hami melon juice

Number	Volatile compound	RI RI_cal_/RI_ref_	Untreated *M* ± *SD* (%)	400 MPa *M* ± *SD* (%)	500 MPa *M* ± *SD* (%)	Qualitative method
Eaters						
1	Methyl acetate	552/559	2.03 ± 0.57	ND	ND	MS/RI
2	Ethyl acetate	612/606	11.09 ± 0.50^a^	30.17 ± 1.5^b^	28.6 ± 1.11^b^	MS/RI
3	Propyl acetate	722/707	1.56 ± 0.42^a^	3.09 ± 0.62^b^	2.76 ± 0.64^b^	MS/RI
4	Methyl butyrate	730/717	1.92 ± 0.49	ND	ND	MS/RI
5	Ethyl 2‐methylpropanoate	762/751	1.57 ± 0.32^a^	1.71 ± 0.02^ab^	2.10 ± 0.16^b^	MS/RI
6	2‐methyl propyl acetate	778/768	8.22 ± 1.87^a^	4.21 ± 1.07^b^	4.91 ± 1.31^b^	MS/RI
7	Methyl 2‐methylbutyrate	786/772	4.93 ± 0.87	ND	ND	MS/RI
8	Ethyl butanoate	808/803	5.69 ± 0.78^a^	5.84 ± 0.13^a^	6.8 ± 0.10^b^	MS/RI
9	Methyl valerate	814/821	0.09 ± 0.02	ND	ND	MS/RI
10	Ethyl 2‐methylbutanoate	816/821	3.40 ± 0.57^a^	4.67 ± 0.46^b^	4.93 ± 0.12^b^	MS/RI
11	2‐methyl butyl acetate	885/877	11.7 ± 2.55^b^	3.45 ± 0.65^a^	4.77 ± 1.24^a^	MS/RI
12	Methyl ethyl thioacetate	976/981	0.05 ± 0.01^a^	2.16 ± 0.38^b^	0.12 ± 0.05^a^	MS/RI
13	Ethyl caproate	996/999	4.83 ± 0.78^b^	1.54 ± 0.44^a^	1.65 ± 0.42^a^	MS/RI
14	3‐hexenol acetate	1,002	ND	0.66 ± 0.16^b^	0.86 ± 0.13^b^	MS
15	Hexyl acetate	1,008/1,011	9.39 ± 0.83^c^	0.17 ± 0.15^a^	2.30 ± 0.49^b^	MS/RI
16	2,3‐butanediol diacetate	1,070/1,064	0.29 ± 0.04^a^	0.20 ± 0.02^b^	0.19 ± 0.03^b^	MS/RI
17	2‐butanol‐2 methyl acetate	1,078	ND	ND	0.1 ± 003	MS
18	Heptyl acetate	1,120/1,111	0.17 ± 0.03	ND	ND	MS/RI
19	Methyl phenylacetate	1,168	3.99 ± 0.16^c^	0.54 ± 0.03^a^	1.29 ± 0.01^b^	MS
20	Dimethyl 2‐methylpropionate	1,193	ND	0.52 ± 0.08^b^	0.46 ± 0.04^b^	MS
21	Butyl butyrate	1,010/994	ND	0.56 ± 0.07^c^	0.4 ± 0.09^b^	MS/RI
22	Diethyl phthalate	1,546	0.15 ± 0.01^a^	0.10 ± 0.03^a^	0.24 ± 0.4^b^	MS
23	Isopropyl palmitate	1,972	0.26 ± 0.05	ND	ND	MS
Alcohols						
24	Ethanol	466	2.01 ± 0.20^a^	5.68 ± 0.13^c^	4.80 ± 0.12^b^	MS
25	(Z)‐3‐hexen‐1‐ol	848/851	4.65 ± 0.47	ND	ND	MS/RI
26	Nonan‐1‐ol	1,164/1,172	0.44 ± 0.07^b^	0.04 ± 0.01^a^	0.07 ± 0.01^a^	MS/RI
27	(Z)‐3‐decen‐1‐ol	1,170	0.46 ± 0.04^a^	0.59 ± 0.05^b^	0.48 ± 0.04^a^	MS
28	(Z)‐6‐nonen‐1‐ol	1,176/1,171	ND	0.46 ± 0.03^b^	0.59 ± 0.06^c^	MS/RI
Aldehydes						
29	Heptanal	896/902	0.17 ± 0.01	ND	ND	MS/RI
30	(E)‐2‐octenal	1,068/1,057	ND	ND	0.12 ± 0.01	MS/RI
31	Decanal	1,202/1,205	ND	0.07 ± 0.01	ND	MS/RI
32	Nonanal	1,110/1,104	0.19 ± 0.01^b^	ND	0.35 ± 0.03^c^	MS/RI
33	(E)‐non‐6‐enal	1,112/1,101	ND	0.39 ± 0.01^b^	0.51 ± 0.04^c^	MS/RI
34	(2E,6Z)‐nona‐2,6‐dienal	1,148/1,155	ND	0.15 ± 0.02^b^	0.15 ± 0.02^b^	MS/RI
35	(E)‐2‐nonenal	1,166/1,162	ND	0.14 ± 0.01^b^	0.13 ± 0.01^b^	MS/RI
Acids						
36	Formic acid	512	2.28 ± 0.07^a^	2.18 ± 0.07^a^	2.28 ± 0.07^a^	MS
37	4‐hydroxybutyric acid	946	0.41 ± 0.03^a^	0.4 ± 0.04^a^	0.42 ± 0.02^a^	MS
38	2‐hydroxy‐4‐methylvaleric acid	1,060	0.08 ± 0.02^a^	0.07 ± 0.01^a^	0.08 ± 0.02^a^	MS
39	Tetradecanoic acid	1,742	0.42 ± 0.06^a^	0.41 ± 0.08^a^	0.4 ± 0.05^a^	MS
Ketones						
40	(5E)‐6,10‐dimethylundeca‐5,9‐dien‐2‐one	1,458/1,448	0.25 ± 0.05^a^	0.23 ± 0.05^a^	0.24 ± 0.03^a^	MS/RI
41	2,2,6‐trimethyl‐3‐butanedione	1,498	0.17 ± 0.03^a^	0.16 ± 0.03^a^	0.16 ± 0.04^a^	MS

Note

RI_cal_: Retention indices calculated of unknown compounds on a DB‐5 capillary column (30 m × 0.25 mm i.d. × 0.25 μm) with a homologous series of n‐alkanes (C4‐C40). RIref: Retention indices obtained from the flavornet database (https://webbook.nist.gov/chemistry/name-ser/) or in the literature. MS: qualitative analysis of mass spectrometry. RI: qualitative analysis of retention index. *M* ± *SD*: Mean ± Standard deviation, *n* = 3. ^abc^Different treatment effects (same kind) for *p* < .05.

Abbreviation: ND, not detected.

Among the volatile components, esters were in majority (Table 2). Many authors have stressed the importance of ester compounds in some fruits (Lambert et al., 1999; Viljanen et al., 2011). Nineteen kinds of esters existed in fresh melon juice, compared to 16 and 17 types of esters for the juice treated at 400 and 500 MPa, respectively. Compared with the untreated sample, the three chemicals of 3‐hexenol acetate, dimethyl 2‐methylpropionate, and butyl butyrate only appeared after HHP of 400 MPa, and four esters (3‐hexenol acetate, 2‐butanol‐2 methyl acetate, dimethyl 2‐methylpropionate, butyl butyrate) only existed after HHP at 500 MPa.

Concurrently, pressure levels caused the disappearance of six esters (methyl acetate, methyl butyrate, methyl 2‐methylbutyrate, methyl valerate, heptyl acetate, and isopropyl palmitate). The HHP‐treated juice still retained the main esters, and 13 identical esters were included in the differently treated samples. As the predominant constituent, ethyl acetate remarkably affected the scent of Hami melons together with ethyl caproate, ethyl 2‐methylbutanoate, ethyl butanoate, and propyl acetate (Ma et al., 2007). Compared with the control, ethyl 2‐methylbutanoate, 2,3‐butanediol diacetate, ethyl acetate, and propyl acetate were increased significantly (*p* < .05) by HHP, whereas no overt difference was noticed between 400 and 500 MPa. It had been reported that alcohols and carboxylic acids could be esterified as volatile esters (Zheng, Zhang, Quan, Zheng, & Xi, 2016). In a previous study, pressure treatments, carried out at 200 and 800 MPa, resulted in increased changes in the concentration of esters (Lambert et al., 1999). The concentrations of methyl phenylacetate, hexyl acetate, ethyl caproate, 2‐methyl butyl acetate, and 2‐methyl propyl acetate were higher in fresh melon samples to HHP‐treated Hami melon juice. The most possible way to ester was to hydrolyze them into corresponding alcohols and acids (Keenan, Brunton, Mitchell, Gormley, & Butler, 2012; Voon, Hamid, Rusul, Osman, & Quek, 2007). Bermejo‐Prada et al. (2015) and Deng et al. (2013) also noticed that some esters declined with increasing pressure, such as dibutyl phthalate, butyl acetate, and 2‐methylbutyl acetate. Conversely, ethyl butanoate, diethyl phthalate, and ethyl 2‐methylpropanoate levels in the control were similar to those detected at 400 MPa (*p* > .05).

Most alcohols found in vegetables and fruits were produced by corresponding aldehydes, which were metabolized by amino acids and fatty acids (Porretta et al., 1995). A new volatile compound, (*Z*)‐6‐nonen‐1‐ol, was found, and (*Z*)‐3‐hexen‐1‐ol disappeared when compared with the raw sample and the pressure‐treated samples. Ethanol and (*Z*)‐3‐decen‐1‐ol were of significantly higher amounts in the 400 MPa juice, compared with the untreated juices, indicating that the amounts of these components increased at elevated pressure. In contrast, the quantity of nonan‐1‐ol was significantly reduced under HHP treatment. In canned tomato pulp products, Porretta et al. (1995) noticed the levels of alcohols decreased after treatment at ultrahigh temperature. In other investigations, HHP treatment markedly modified the volatile odor compounds of several juices (Santhirasegaram, Razali, George, & Somasundram, 2015; Yi et al., 2017; Zheng et al., 2016). In this study, pressure had the greatest effects on the type and content of aldehydes. Decanal, (*E*)‐2‐octenal, (*E*)‐6‐nonenal, (2*E*,6*Z*)‐nona‐2,6‐dienal, and (*E*)‐2‐nonenal were absent from untreated juice. These changes would influence fresh Hami melon odor. For example, the thresholds of (2*E*,6*Z*)‐nona‐2,6‐dienal, (*E*)‐6‐nonenal, and (*Z*)‐6‐nonen‐1‐ol were low, which contribute to the cucumber‐like, green odor (Ortizserrano & Gil, 2010; Tangwongchai, Ledward, & Ames, 2000). Thus, HHP treatment resulted in a stronger fragrant odor of the samples than the fresh one. Compared with the different pressures, the refreshing and fresh odors under the 400 MPa‐treated Hami melon juice were less obvious than the treated one by 500 MPa. (*E*)‐2‐Octenal was only detected at 500 MPa, whereas decanal was only detected at 400 MPa. (*E*)‐2‐Octenal exhibited an oily odor at a high concentration, which might adversely affect the aroma of Hami melon juice.

**Figure 1:**
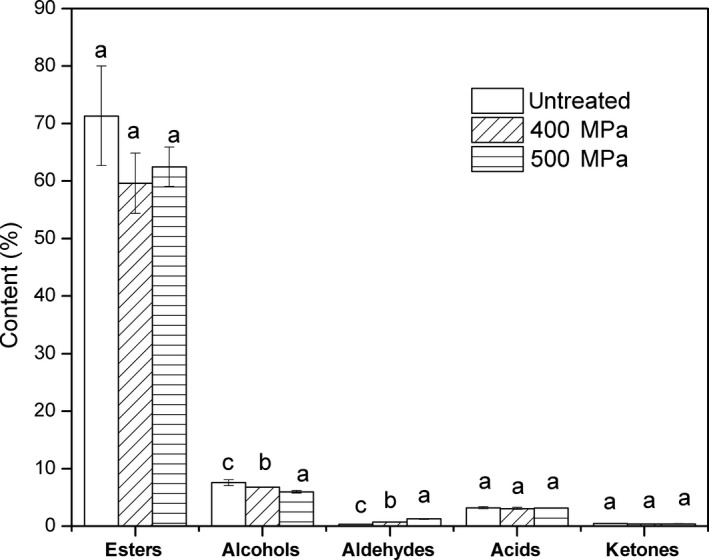
Change of esters, alcohols, aldehydes, acids, and ketones

Figure 1 showed the content changes in esters, alcohols, aldehydes, acids, and ketones of untreated, 400, and 500 MPa‐treated samples, respectively. Among the five groups of compounds, esters presented the most contents (59.59%–71.34%), followed by alcohols (5.95%–7.56%), acids (3.05%–3.2%), aldehydes (0.36%–1.25%), and ketones (0.39%–0.42%). Pang et al. (2012) also reported similar findings in the study on Jiashi muskmelon juice about determining the active compounds of aroma through GC‐MS‐olfactometry. Similar to previous study, Gonda et al. (2010) found that the aroma of different melon fruits was mainly composed of various esters with floral and fruity flavors, and aldehydes and alcohols with faint‐scent and grassy odors. The observed result showed that esters, acids, and ketones were not significantly changed by the different treatments. When compared to the nontreated sample, the samples exposed to HHP treatments showed evidently lower and higher alcohol and aldehyde contents, respectively (*p* < .05). Earlier studies showed that under the effects of pressure, the content of aldehydes in juice was increased (Liu et al., 2014; Viljanen et al., 2011). From the perspective of flavor, it is well known that the most abundant volatiles may not be the most significant sensory elements. Some volatiles, even those at low concentrations, which were often viewed as crucial flavor compounds, play a decisive role in the aroma perceived (Bermejo‐Prada et al., 2015). Although ultra‐high‐pressure treatment slightly increased the cucumber‐like odor, HHP‐treated melon juice still showed its unique Hami melon flavor.

**Figure 2:**
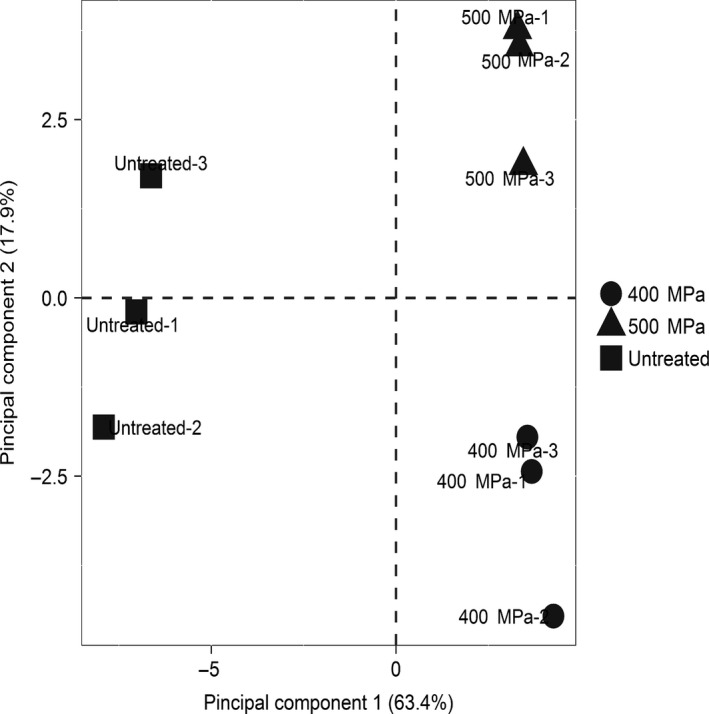
Principal component analysis (PCA) plot of aroma compounds of Hami melon juice at different treatments

Figure 2 provided a PCA, which displayed the relation between different processing conditions and aroma compounds. By extracting the main components, the aroma components were confined to the first two principal components (PC1 and PC2, respectively), which both explained more than 80% (63.4% and 17.9%, respectively) of the data variance. Thus, the data interpretation rate was good. It could be seen from the distribution in the PCA loading plot that the 400 and 500 MPa were separated from the untreated group by a large distance, indicating a significant difference between the control group and the HHP groups in the content and type of the overall aroma components.

### 3.2 Change in amino acid contents

Table 3 showed the changes in amino acids of Hami melon juice under the different treatment conditions. It was known that amino acids differed in their sensitivity to the processing conditions, depending on the processing methods and materials (Boye, Wijesinhabettoni, & Burlingame, 2012). The main free amino acids of the Hami melon juice in this study included arginine (Arg), lysine (Lys), phenylalanine (Phe), leucine (Leu), isoleucine (Iso), valine (Val), alanine (Ala), glycine (Gly), proline (Pro), glutamate (Glu), serine (Ser), threonine (Thr), and aspartic acid (Asp). For all these amino acids, six amino acids were essential and the rest seven were nonessential amino acids. According to Table 3, there were many kinds of amino acids in Hami melon juice, but the total content was low. The concentration of total amino acids increased greatly after HHP treatment, compared to the samples without treatment (*p* < .05). Lys was absent from the untreated sample, and Iso was found only after HHP treatment at 400 MPa. In comparison with the control, the content of each amino acid in the 400 MPa‐treated juice increased, except Pro, which did not change. When the pressure rose from 400 to 500 MPa, Asp and Leu were evidently increased while Thr, Ser, and Arg decreased (*p* < .05), and Glu, Gly, Ala, Val, and Phe had no obvious change. Seven‐day‐old Brussel sprout seedlings were exposed to HHP treatment up at 800 MPa for 3 min, and Barba, Poojary, Wang, Olsen, and Orlien (2017) identified slight effects on the concentrations of 10 free amino acids (Tyr, Trp, Ser, Pro, Phe, Leu, Gly, Glu, Asp, and Ala). However, their effects played a key role. The reason for the increase in amino acids might be that HHP treatment caused proteolysis (Dos‐Santos et al., 2013; Shigematsu et al., 2010). Thr, Ser, and Arg accounted for less amount at 500 MPa, due to the proteolytic conversion of those amino acids (Martínez‐Monteagudo & Balasubramaniam, 2016). In another study, Wang et al. (2016) confirmed that HHP treatment might affect physical and chemical reactions, including the amino acids content, due to synergy or antagonism between different components.

**Table 3:** The changes of amino acids in Hami melon juice

Amino acids	Untreated *M* ± *SD*	400 MPa *M* ± *SD*	500 MPa *M* ± *SD*
Aspartic acid	0.047 ± 0.001^a^	0.060 ± 0.002^c^	0.056 ± 0.001^b^
Threonine	0.012 ± 0.001^a^	0.015 ± 0.001^b^	0.012 ± 0.001^a^
Serine	0.019 ± 0.002^a^	0.023 ± 0.001^b^	0.019 ± 0.002^a^
Glutamate	0.11 ± 0.006^a^	0.15 ± 0.006^b^	0.14 ± 0.015^b^
Proline	0.010 ± 0.001^a^	0.012 ± 0.001^a^	0.012 ± 0.002^a^
Glycine	0.012 ± 0.002^a^	0.016 ± 0^b^	0.016 ± 0.001^b^
Alanine	0.075 ± 0.005^a^	0.097 ± 0.002^b^	0.098 ± 0.002^b^
Valine	0.016 ± 0.001^a^	0.020 ± 0.001^b^	0.021 ± 0.002^b^
Isoleucine	ND	0.012 ± 0.001	ND
Leucine	0.011 ± 0.001^a^	0.014 ± 0.001^b^	0.013 ± 0.002^ab^
Phenylalanine	0.010 ± 0.002^a^	0.016 ± 0.001^b^	0.016 ± 0.001^b^
Lysine	ND	0.011 ± 0.001^b^	0.011 ± 0.002^b^
Arginine	0.022 ± 0.001^a^	0.025 ± 0.002^b^	0.022 ± 0.002^a^
Total amino acids	0.34 ± 0.01^a^	0.47 ± 0.01^c^	0.44 ± 0.02^b^

Note

*M* ± *SD*: Mean ± Standard deviation, *n* = 3. ^abc^Different treatment effects (same kind) for *p* < .05.

Abbreviation: ND, not detected.

### 3.3 Change in fatty acids

The fatty acids of palmitic, palmitoleic, linoleic, α‐linolenic, and oleic acids were the main fatty acid components in Hami melon juice, and they were key precursor substances of aroma esters (Chen et al., 2013). As shown in Table 4, after the fresh melon juice was treated at 400 MPa, palmitic, linoleic, and α‐linolenic acids all showed a decreasing trend (*p* < .05) while oleic acid and palmitoleic acid have nonsignificant change. After HHP treatment at 500 MPa, however, the concentrations of all five fatty acids mentioned above decreased apparently compared with HHP at 400 MPa. During ultra‐high‐pressure treatment, it was reported that free fatty acids were mainly oxidized to form *n*‐hexanal and hexenal (Porretta et al., 1995). Similar reports were seen in other experiments (Aganovic et al., 2014; Liu et al., 2014).

**Table 4:** The changes of fatty acids in Hami melon juice

Fatty acids	Untreated *M* ± *SD*	400 MPa *M* ± *SD*	500 MPa *M* ± *SD*
Palmitic acid	20.53 ± 0.35^c^	12.47 ± 0.35^b^	11.20 ± 0.1^a^
Palmoleic acid	4.08 ± 0.22^b^	3.93 ± 0.06^b^	2.82 ± 0.07^a^
Oleic	4.82 ± 0.13^b^	4.75 ± 0.25^b^	4.35 ± 0.06^a^
Linoleic	20.43 ± 0.66^c^	14.80 ± 0.95^b^	10.8 ± 0.6^a^
α‐linolenic acid	10.67 ± 0.45^c^	6.38 ± 0.28^b^	4.86 ± 0.13^a^

Note

*M* ± *SD*: Mean ± Standard deviation, *n* = 3. ^abc^Different treatment effects (same kind) for *p* < .05.

### 3.4 Correlation analysis of aroma components with free amino acids

**Figure 3:**
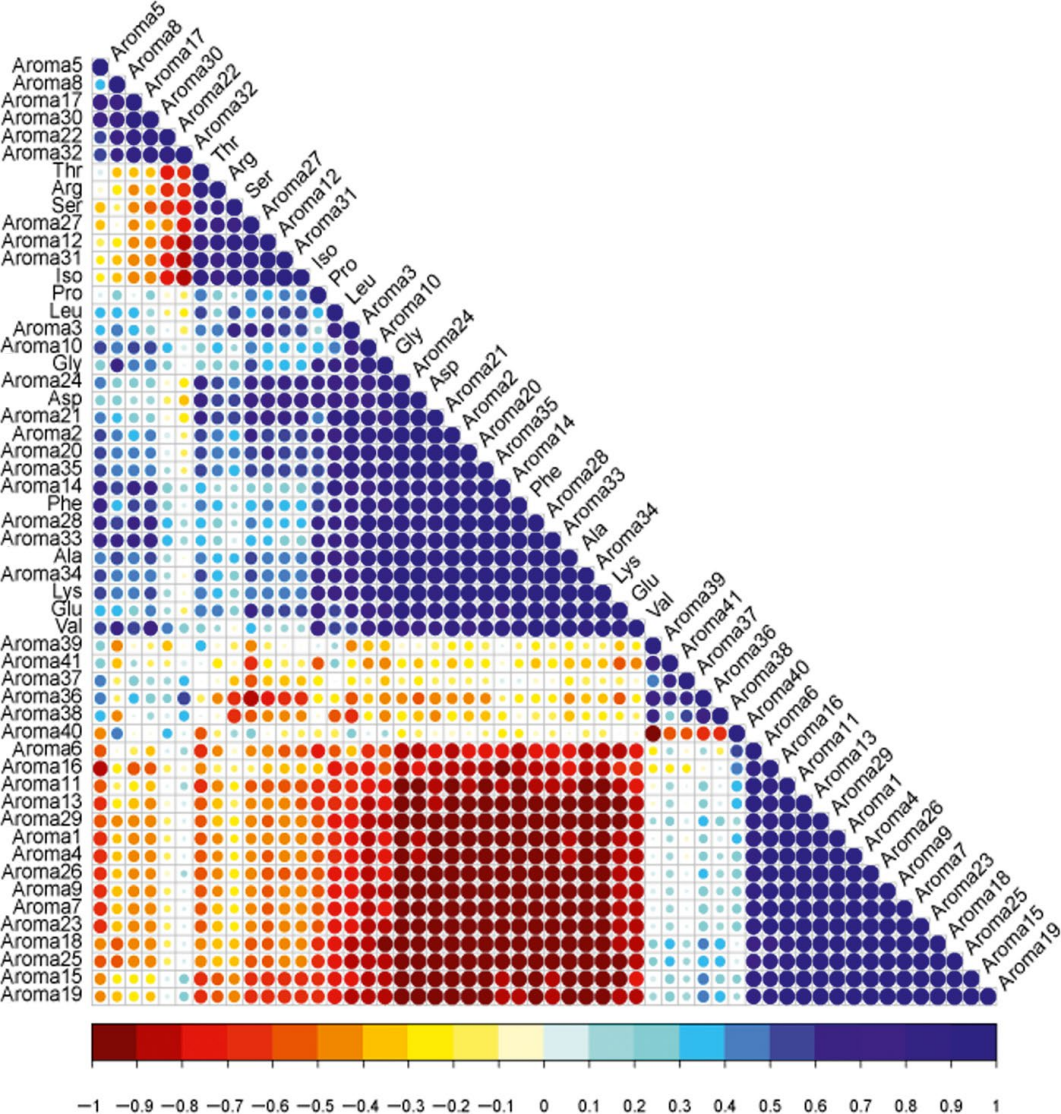
Correlation analysis of aroma components and amino acids

Apart from an attractive color, various amino acids could produce different tastes, like umami, bitter, and sweet (Tian et al., 2016). Thus, if the content of amino acids was changed, the aroma substance and the quality of Hami melon juice might be affected. Figure 3 showed a bitmap of the correlation analysis between the aroma components and amino acids. The darker the color was, the higher the correlation coefficient was. Red and blue represented the negative correlation and positive correlation, respectively. The number of aroma substances in Figure 3 corresponded to that in Table 2.

As listed in Figure 3, Iso and Leu had a great positive correlation with decanal and methyl ethyl thioacetate. Gly had a notable positive correlation with ethyl butanoate, ethyl 2‐methylbutanoate, propyl acetate, and (*Z*)‐3‐decen‐1‐ol. The change in Asp was also clearly positively correlated with the altered levels of ethanol, propyl acetate, ethyl 2‐methylbutanoate, decanal, methyl ethyl thioacetate, and (*Z*)‐3‐decen‐1‐ol. Phe had a distinct positive correlation with (*E*)‐2‐nonenal, dimethyl‐2‐methylpropionate, ethyl acetate, ethanol, and ethyl 2‐methylbutanoate. Ala and several volatile compounds ((*E*)‐6‐nonenal, 2‐hydroxy‐4‐methylvaleric acid, 3‐hexenol acetate, (*E*)‐2‐nonenal, dimethyl 2‐methylpropionate, ethanol) showed a evident positive correlation. Lys and Glu also had an obvious positive correlation with many aroma components, such as (2*E*,6*Z*)‐nona‐2,6‐dienal, (*E*)‐6‐nonenal, (*Z*)‐6‐nonen‐1‐ol, 3‐hexenol acetate, (*E*)‐2‐nonenal, dimethyl‐2‐methylpropionate, and ethanol. In comparison with these correlations mentioned, a relatively stronger positive correlation existed between Val and some independent volatiles, like (*Z*)‐6‐nonen‐1‐ol. The amino acids were positively associated with some esters, C9 aldehydes, and C9 alcohols, such as ethyl acetate, ethyl 2‐methylbutanoate, methyl ethyl thioacetate, dimethyl 2‐methylpropionate, (*E*)‐6‐nonenal, (*E*)‐2‐nonenal, and (*Z*)‐6‐nonen‐1‐ol. Thr, Arg, Ser, and Iso had highly negative correlation with nonanal and diethyl phthalate.

From the dark red area in the lower part of Figure 3, it could be seen that many aroma components (e.g., 2‐methyl propyl acetate, 2,3‐butanediol diacetate, 2‐methyl butyl acetate, ethyl caproate, heptanal, methyl acetate, and nonan‐1‐ol) were negatively correlated with Asp, Phe, Ala, Lys, and Val. Among these substances, there were two alcohols (nonan‐1‐ol and (*Z*)‐3‐hexen‐1‐ol), 2 aldehydes (heptanal and nonanal), and 12 esters (e.g., hexyl acetate, heptyl acetate, methyl phenylacetate, ethyl caproate, and 2‐methyl butyl acetate). In the research undertaken by Dos‐Santos et al. (2013), the closest correlation was found in the volatiles and Phe, Leu, Val, Iso, or methionine. Zhang et al. (2018) noted that many amino acids (e.g., Ala) were closely related to aldehydes, pyrazine, and pyrrole.

### 3.5 Correlation analysis of aroma components with fatty acids

**Figure 4:**
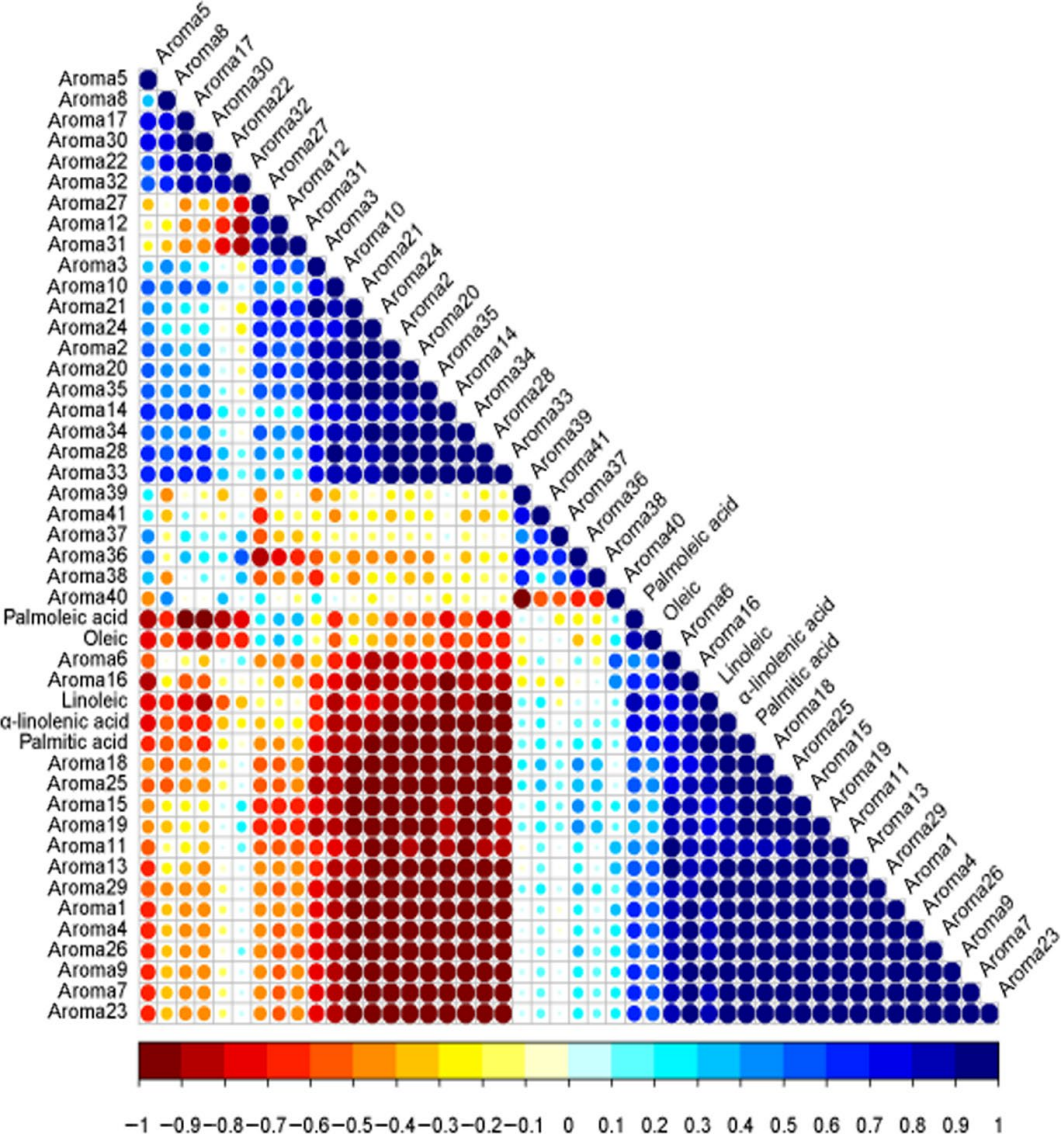
Correlation analysis of aroma components and fatty acids

Fatty acids were usually considered to be closely related to some odors (Braddock & Kesterson, 1973; Liu et al., 2014; Shi et al., 2018). Figure 4 illustrated the relationship between five kinds of fatty acids and 41 aroma substances. Palmoleic acid and oleic acid had similar correlations with the aroma components. The concentrations of palmitoleic acid and oleic acid were negatively correlated with ethyl 2‐methylpropanoate, ethyl butanoate, 2‐butanol‐2 methyl acetate, (*E*)‐2‐octenal, nonanal, 3‐hexenol acetate, (2*E*,6*Z*)‐nona‐2,6‐dienal, (*Z*)‐6‐nonen‐1‐ol, and (*E*)‐6‐nonenal. On the contrary, they were positively correlated with 2,3‐butanediol diacetate, ethyl caproate, heptanal, methyl acetate, methyl butyrate, nonan‐1‐ol, methyl valerate, and methyl 2‐methylbutyrate.

The relationships between the remaining three fatty acids and the aroma were also similar to each other. Palmitic acid, linoleic acid, and α‐linolenic acid had a strong positive correlation with many aroma components in the lower part of Figure 4 (e.g., heptyl acetate, (*Z*)‐3‐hexen‐1‐ol, heptanal, methyl acetate, methyl butyrate, methyl valerate, isopropyl palmitate, ethyl caproate). The aroma substances were negatively associated with palmitic acid, linoleic acid, and α‐linolenic acid included, involving ethyl 2‐methylpropanoate, ethyl butanoate, 2‐butanol‐2 methyl acetate, (*E*)‐2‐octenal, propyl acetate, ethyl 2‐methylbutanoate, butyl butyrate, ethyl acetate, (*E*)‐2‐nonenal, and 3‐hexenol acetate. The five fatty acids were found to be primarily associated with esters and aldehydes. Previous report drew similar conclusions. For instance, Viljanen et al. (2011) found that compared with standard tomatoes, the content of linolenic acids in the neutral lipid fraction of cherry tomatoes was higher, possibly leading to a higher concentration of (*E*)‐2‐hexenal and hexanal. Takahashi and Goto‐Yamamoto (2011) found the content of medium‐chain fatty acids was correlated with ethyl hexanoate concentrations in *moromi* (a fermented blend of barley, barley koji, yeast, and water), and hexanoic acid was correlated with fatty odor.

The results of the current investigation demonstrated esters and aldehydes were important aroma substances in Hami melon juice, and the levels of esters and aldehydes were closely associated with those of the amino acids and fatty acids, which provided a good basis for preserving the aroma of Hami melon drinks.

## 4 CONCLUSIONS

The high‐pressure treatment impacted on certain volatile odor compounds. The PCA diagram of aroma confirmed this conclusion. Thirty‐one volatile compounds in total compounds were identified in fresh juice, whereas 30 and 32 volatile compounds were identified in treated Hami melon juices under 400 and 500 MPa HHP, respectively. Compared with the control, the type and content of aldehydes, esters, and alcohols in the HHP Hami melon juice were changed, but ketones and acids did not get changed. However, only the aldehydes were clearly changed, when compared 400 MPa with 500 MPa. The increase in aldehydes renders the cucumber‐like odor apparent, while unique Hami melon flavor was reduced. HHP treatment had effects on the 12 FAA concentrations (Asp, Thr, Ala, Glu, Gly, Leu, Phe, Ser, Arg, Val, Lys, and Iso). Compared with fresh melon juice, the five fatty acids presented a evident downward trend at a pressure of 500 MPa. At 400 MPa, only palmitic acid, oleic acid, and linolenic acid were decreased. The correlation analysis of amino acids and fatty acids on aroma substances showed that amino acids were mainly related to 12 esters (hexyl acetate, heptyl acetate, methyl phenylacetate, ethyl caproate, 2‐methyl butyl acetate, among others) and two alcohols, namely (*Z*)‐3‐hexen‐1‐ol and nonan‐1‐ol, and 2 aldehydes (heptanal, nonanal), whereas the fatty acids were mainly related to some esters and aldehydes, such as ethyl 2‐methylpropanoate, ethyl butanoate, 2,3‐butanediol diacetate, 2‐butanol‐2 methyl acetate, heptanal, and (*E*)‐non‐6‐enal. The above analysis showed that the flavor of the 400 MPa sample was better than the 500 MPa one.

## CONFLICT OF INTEREST

The authors declare no conflict of interest.

## ETHICAL APPROVAL

This study does not involve neither human nor animal testing.
